# Incidence and complications of interstitial lung disease in users of tocilizumab, rituximab, abatacept and anti-tumor necrosis factor α agents, a retrospective cohort study

**DOI:** 10.1186/s13075-015-0835-7

**Published:** 2015-11-11

**Authors:** Jeffrey R. Curtis, Khaled Sarsour, Pavel Napalkov, Laurie A. Costa, Kathy L. Schulman

**Affiliations:** Division of Clinical Immunology and Rheumatology, UAB Arthritis Clinical Intervention Program, University of Alabama at Birmingham, FOT 802D, 510 20th Street South, Birmingham, AL 35924 USA; Genentech, 1 DNA Way, South San Francisco, CA 94080 USA; Outcomes Research Solutions, Inc., 303 Wyman Street, Waltham, MA 02451 USA

**Keywords:** Rheumatoid arthritis, Interstitial lung disease, Biologics, Hospitalization

## Abstract

**Introduction:**

Interstitial lung disease (ILD) is a common extra-articular condition in rheumatoid arthritis (RA), but few studies have systematically investigated its incidence and risk factors in patients receiving anti-tumor necrosis factor-alpha (anti-TNFα) agents or alternate mechanisms of action (MOAs) (e.g., T-cell, B-cell, and interleukin-6 inhibitors).

**Methods:**

RA patients at least 18 years old were selected from the MarketScan databases (2010–2012) if they had at least one prescription/administration of abatacept, rituximab, tocilizumab, or anti-TNF after having discontinued a different biologic agent and meeting enrollment criteria. Cox models estimated the risk of incident ILD and ILD-related hospitalization. Sensitivity analyses used an alternate ILD case definition.

**Results:**

We identified 13,795 episodes of biologic exposure in 11,219 patients. Mean (standard deviation) follow-up was 0.7 (0.5) years. Patients receiving alternate MOA agents were more likely to have had recent exposure to steroids, prior exposure to a greater number of biologics, and history of ILD, anemia, chronic obstructive pulmonary disease, and other pulmonary conditions. When the sensitive definition was used, unadjusted ILD incidence rates (95 % confidence interval, or CI) ranged from 4.0 (1.6–8.2, abatacept) to 12.2 (5.6–23.2, infliximab) per 1000 person-years. Being older (hazard ratio (HR) 3.5; 95 % CI 2.1–6.0), being male (HR 3.1; 95 % CI 1.2–8.4), and having another pulmonary condition (HR 4.8; 95 % CI 1.7–13.7) were associated with increased ILD incidence in either sensitive and/or specific models. There were no significant differences by biologic class. Hospitalization rates (95 % CI) when the sensitive definition was used ranged from 55.6 (6.7–200.7, tocilizumab) to 262.5 (71.5–672.2, infliximab). In Cox models, recent methotrexate exposure was associated with reduced ILD hospitalization (HR 0.16; 95 % CI 0.06–0.46), whereas being male (HR 2.5; 95 % CI 1.3–4.8) and having had a hospitalization for asthma (HR 3.4; 95 % CI 1.2–9.8) or ILD/pneumonia (HR 2.3; 95 % CI 1.1–4.7) in the 12 months prior to index were associated with increased hospitalization risk.

**Conclusions:**

There were no significant differences in the risk of ILD and its related complications between RA patients receiving anti-TNFα agents and those receiving alternate MOA agents. Further studies are needed that account for differences in baseline characteristics in order to fully evaluate the risk of ILD and its complications.

## Introduction

Roughly half of patients with rheumatoid arthritis (RA) will have some form of extra-articular involvement, such as interstitial lung disease (ILD) [1–3]. ILD refers to a collection of lung disorders classified together because they all affect the tissue and space around the alveoli called the interstitium. Depending on the specific disease in question, the alveoli, airways, blood vessels, and pleura may also be affected. Manifestations of ILD include respiratory symptoms (e.g., dyspnea and non-productive cough), specific chest radiographic abnormalities, decreased lung volume, and microscopic patterns of inflammation and fibrosis [4]. Although the condition is heterogeneous in RA, the majority of cases are similar to non-specific interstitial pneumonia and usual interstitial pneumonia. The prevalence of ILD in patients with RA varies from 5 % to 58 %, depending on the ILD case definition and RA severity in the population studied [5–7]. The 1-year incidence of ILD has been reported at 2.8 % [8]. Symptoms may be subtle or non-existent at onset, despite patients having radiographic features consistent with ILD.

Cigarette smoking and high levels of circulating rheumatoid factor and anti-cyclic citrullinated peptides have been identified as risk factors for ILD. Drug-induced ILD has been reported in the past as a rare but severe adverse event associated with a number of agents used to treat RA, namely non-steroidal anti-inflammatory drugs, intravenous immunoglobulin, gold, methotrexate, leflunomide, and cyclophosphamide [9–13]. New-onset ILD or ILD worsening has also been reported as a possible consequence of biologic agents, including three of the most widely used anti-tumor necrosis factor alpha (anti-TNFα) inhibitors (infliximab, etanercept, and adalimumab) [12–16]. However, these associations are frequently based on case reports, in differing patient populations with multiple medication exposures and using various definitions of ILD.

It has been hypothesized that biologic therapy might cause serious respiratory events by inducing idiosyncratic reactions, accelerating pre-existing ILD, modifying ILD into a more injurious phenotype, or increasing susceptibility to infection, yet the precise mechanism of action (MOA) is unknown [17, 18]. These agents may also have a therapeutic effect in RA-ILD since inflammatory cytokines, including TNF, are elevated in patients with idiopathic pulmonary fibrosis. Studies have been conducted to assess the benefit of these agents as therapy for ILD. One study demonstrated a non-statistically significant reduction in ILD progression, and a second trial is still under way [17].

The present study evaluated ILD incidence and exacerbation among users of abatacept (T-cell inhibitor), rituximab (B-cell inhibitor), and tocilizumab (interleukin-6 (IL-6) inhibitor) compared with anti-TNFα agents in a cohort of adult RA patients who previously had exposure to at least one biologic therapy.

## Methods

### Data source

The data source for this retrospective cohort study was the MarketScan Commercial Claims and Encounters (Commercial) and the Medicare Supplemental and Coordination of Benefit (Medicare) databases. Both databases contain de-identified health insurance enrollment information and claims data for inpatient and outpatient medical services as well as outpatient prescriptions. The commercial database includes employees, spouses, and dependents covered by employer-sponsored private health insurance. The Medicare database profiles the health-care experience of retirees with Medicare supplemental insurance paid by employers. All patient data used in this analysis were de-identified in compliance with the Health Insurance Portability and Accountability Act and as such did not require institutional review board approval.

### Patient population

RA patients, at least 18 years old, were selected into the study provided that they had a prescription or administration of a new biologic agent between 1 January 2010 and 30 June 2012, evidence of having previously discontinued a different biologic agent at any time in the past, and at least one diagnosis of RA (ICD-9-CM 714.0, 714.3) on a non-diagnostic claim either during the baseline period or within the first 30 days of follow-up. Prior biologic use was required in order to homogenize the patient population to those individuals with more refractory disease who had received biologics in the past and to allow better comparability with patients using second- and third-line agents. The study index date was the date of first prescription/administration of the biologic agent for which the patient met all eligibility requirements. Patients were required to be continuously enrolled for at least 12 months prior to index and to have had both medical and pharmacy benefit plus complete data availability during both baseline and follow-up periods. Patients with a history of malignancy (ICD-9-CM 140–171, 174–209, 230–234), ulcerative colitis (ICD-9-CM 556.xx), psoriatic arthritis (ICD-9-CM 696.0x), Crohn disease (ICD-9-CM 555.xx), psoriasis (ICD-9-CM 696.1x), or ankylosing spondylitis (ICD-9-CM 720.xx) during the 12 months prior to index were excluded from the study. Patients with baseline evidence of prior use of the qualifying biologic were also deemed study-ineligible.

Each patient was permitted to contribute multiple episodes to the analytic data set. Patients were followed within each treatment episode until disenrollment from MarketScan, end of the study period, discontinuation of the qualifying biologic, addition of a new biologic agent, onset of any malignancy, or the primary study outcome, incident ILD. A second endpoint, the occurrence of an ILD complication, was examined in a separate analysis in a cohort of RA patients with a history of ILD.

### Cohort definitions

Treatment episodes were constructed for each individual biologic agent by computing drug exposure on each day of the study. Pharmacy-based exposure was based on the date of fill and number of days of supply associated with each prescription. For provider-administered injectable or infused medications, exposure was based on the administration date and a clinically relevant exposure window. Administrations of etanercept were assigned a 7-day coverage window, whereas adalimumab and certolizumab were assigned a 14-day coverage window. Administration of tocilizumab and abatacept was assigned a 28-day coverage window, rituximab 183 days, infliximab 56 days, and injectable corticosteroids a 28-day coverage window. To capture events that might have occurred shortly after discontinuation while an agent’s effects were still material and to address changes in medication instruction that may have occurred after the issuance of the original prescription, a fixed grace period of 90 days was applied to all claims, pharmacy-issued or physician/facility-administered. Sensitivity analyses that expanded the grace period from 90 to 120 days were conducted.

Health-care providers obtain reimbursement for medications by using Healthcare Common Procedure Coding System (HCPCS) codes. Claims for newly licensed medications use a non-specific HCPCS code (e.g., J3490 and J3590) until a unique HCPCS code specific to each drug is assigned, usually 1–2 years post-launch. We adapted a validated algorithm [19] in order to identify both tocilizumab and certolizumab pegol claims from the pool of unclassified drugs/biologics claims. This algorithm, developed by using Medicare data linked to an arthritis registry, had good performance characteristics: sensitivity of 94 % (95 % confidence interval (CI) 80 %–99 %), specificity of 100 % (95 % CI 99 %–100 %), and positive predictive value of 97 % (95 % CI 84 %–100 %) [20].

Treatment episodes were constructed at the level of each biologic agent, but for reporting purposes, exposure was collapsed into one of four groups: tocilizumab, abatacept, rituximab, and all anti-TNFα agents in aggregate. Only ILD incidence and complication rates were presented separately by individual anti-TNF agent.

### Study outcomes

The primary study outcome was the incidence of ILD. Given the well-documented challenges in ILD case ascertainment, especially in claims-based data, we defined ILD both conservatively, using a more specific definition, as well as more broadly, in order to maximize sensitivity [21]. The two definitions differed in the ICD-9-CM diagnosis (Appendix), the position of the ILD diagnosis on the claim, and the presence of an eligible ILD diagnostic test (computed tomography (CT) scan of the thorax or lung biopsy) within 90 days of the ILD diagnosis. Definition 1, the more specific definition, included diagnoses of post-inflammatory pulmonary fibrosis and idiopathic interstitial pneumonia, required that diagnoses on inpatient claims be in the primary position, and required that the ILD diagnostic test occur in the 90 days prior to diagnosis. In contrast, definition 2, the more sensitive definition, included diagnoses of rheumatoid lung and other specified and unspecified alveolar and parietoalveolar pneumonopathies, accepted these diagnoses in any claim position, and did not require evidence of a preceding ILD diagnostic test.

The second study outcome was the frequency of hospitalizations for ILD or an ILD-related complication in patients with a baseline history of ILD. Eligible hospitalizations included hospitalization with a primary diagnosis of ILD, pneumonia (ICD-9-CM 480.xx-486.xx, 487.0x), or lung transplant. Hospitalization was chosen as a proxy of ILD-related exacerbations or complications. Only the first event was measured and evaluated since the duration of ILD exacerbation varies.

### Statistical analysis

Descriptive results were produced twice: once with the more specific definition of ILD and a second time with the more sensitive definition. Basic analyses included descriptive profiles of all independent and dependent variables. Categorical variables were summarized in frequency tables. Continuous and other numerical variables were summarized by the number of observations, means, standard deviations (SDs), and medians. Statistical tests of significance for differences in these distributions were carried out with chi-squared tests used to assess the statistical significance of categorical variables; *t* tests and analysis of variance were used for continuous variables. Generalized estimating equations were used to adjust for clustering.

The number and proportion of patients with each event (incident ILD and ILD-related hospitalization), as well as the rate per 1000 person-years (PY) of observation, were reported. Cox proportional hazards models were developed to assess the relative hazard of each event during follow-up, adjusting for differences in baseline characteristics among the biologic exposure groups. The relative hazard of ILD incidence was estimated as a function of age group (<65 years reference), study cohort (anti-TNF cohort reference), gender (female reference), recent glucocorticoid or methotrexate exposure defined as exposure in the 6 months prior to index, or baseline history of a pulmonary condition—chronic obstructive pulmonary disease (COPD), asthma, or pneumonia—other than ILD. In the assessment of ILD complications among patients with a history of ILD, only one Cox proportional hazards model could be constructed because of small event counts, estimated as a function of age group (<65 years reference), study cohort (anti-TNF cohort reference), gender (female reference), recent glucocorticoid or methotrexate exposure defined as exposure in the 6 months prior to index, or a recent hospitalization with a diagnosis of asthma, COPD, ILD, or pneumonia. The latter variables were used as proxies for the severity or complexity of ILD.

## Results

**Fig. 1:**
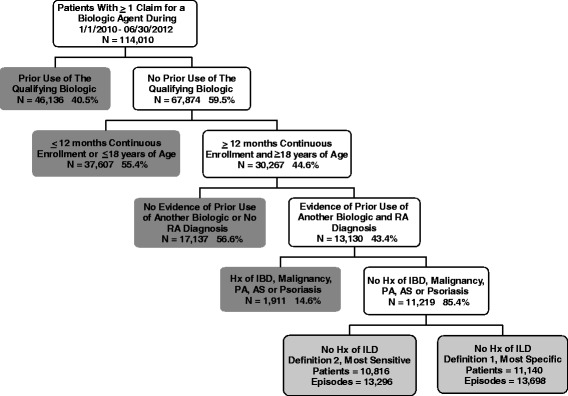
Application of selection criteria and patient disposition. *AS* ankylosing spondylitis, *Hx* history, *IBD* inflammatory bowel disease, *ILD* interstitial lung disease, *PA* psoriatic arthritis, *RA* rheumatoid arthritis

There were 114,010 patients in the MarketScan data extract, with 1.2 million claims for a biologic medication during the case selection window (1 January 2010 through 30 June 2012). Sixty percent of these patients (n = 67,874) were classified as “new” users since they had no evidence of prior exposure to the qualifying biologic using all available claims data (2001–2012) during the period prior to their 79,525 potentially eligible episodes. Fewer than half of these patients (44.6 %; n = 30,267) met age-eligible (at least 18 years) and benefit-eligible continuous enrollment criteria (at least 12 months prior to index), and only half of patients meeting enrollment and age criteria had evidence of prior use of any other biologic (49 %; n = 14,681). An additional 23 % of patients in total were deemed ineligible because of other criteria (no RA diagnosis, clinical history of psoriatic arthritis, ankylosing spondylitis, cancer, inflammatory bowel disease, or psoriasis). Once the eligible study population was identified, patients were segmented into those with (499 episodes and 419 patients) and without (13,296 episodes and 10,800 patients) a history of ILD. Those patients who did not have a history of prevalent ILD were eligible for the ILD incidence study, whereas those with a history were eligible for the complications portion of the study. Figure 1 depicts accrual graphically.

### Patient characteristics

#### Patients without a history of ILD

Despite differences in the ILD detection rate associated with each ILD definition, there was little variability in patient characteristics. These characteristics, presented in tabular form in Table 1 and summarized below, were based on the most sensitive ILD definition. Patients were exposed on average for 0.7 years (8.3 months), with little variation across the biologic exposure group.

**Table 1:** Baseline demographics and clinical characteristics: patients without ILD history using the most sensitive ILD definition

	New anti-TNF Treatment episodes	New tocilizumab Treatment episodes	New rituximab Treatment episodes	New abatacept Treatment episodes
	N = 7951	N = 1528	N = 1134	N = 2683
Female, n (%)^a^	6462 (81.3)	1267 (82.9)	931 (82.1)	2228 (83.0)
Age, mean (SD)^a^	51.7 (12.5)	53.8 (12.0)	53.8 (12.1)	53.9 (12.6)
Geographic region, n (%)^a^				
Northeast	1285 (16.2)	230 (15.1)	172 (15.2)	393 (14.6)
North Central	1866 (23.5)	382 (25.0)	306 (27.0)	686 (25.6)
South	3212 (40.4)	583 (38.2)	411 (36.2)	1100 (41.0)
West	1536 (19.3)	323 (21.1)	235 (20.7)	481 (17.9)
Rural indicator, n (%)^a, b^	1315 (16.5)	225 (14.7)	216 (19.0)	392 (14.6)
Medicare primary payer, n (%)^a, b^	1001 (12.6)	245 (16.0)	195 (17.2)	488 (18.2)
Follow-up person years per patient, mean (SD)	0.7 (0.6)	0.7 (0.5)	0.7 (0.5)	0.7 (0.5)
No of prior biologics, mean (SD)^b, c^	1.4 (0.7)	2.1 (1.1)	1.9 (0.9)	1.6 (0.8)
Prior exposure to ≥2 biologics	990 (12.5)	715 (46.8)	334 (29.5)	198 (7.4)
Prior exposure to ≥3 biologics	186 (2.3)	136 (8.9)	40 (3.5)	4 (0.1)
RA medication history, n (%)^d^				
Any anti-TNFα agent^b^	5532 (69.6)	617(40.4)	571 (50.4)	1865 (69.5)
Methotrexate	4293 (54.0)	785 (51.4)	604 (53.3)	1361 (50.7)
All other DMARDs	2575 (32.4)	531 (34.8)	410 (36.2)	883 (32.9)
Prescription NSAIDs	3407 (42.8)	635 (41.6)	449 (39.6)	1080 (40.3)
Glucocorticoid daily dosage, mean (SD)^e^				
None^b^	2307 (29.0)	299 (19.6)	214 (18.9)	599 (22.3)
<7.5 mg/day	4355 (54.8)	864 (56.5)	642 (56.6)	1534 (57.2)
≥7.5 mg/day^b^	1285 (16.2)	362 (23.7)	278 (24.5)	549 (20.5)
Comorbid condition, n (%)^f^				
Anemia^b^	163 (2.1)	45 (2.9)	29 (2.6)	52 (1.9)
Asthma	294 (3.7)	76 (5.0)	47 (4.1)	118 (4.4)
Cerebrovascular disease	131 (1.6)	20 (1.3)	21 (1.9)	44 (1.6)
COPD^b^	209 (2.6)	59 (3.9)	40 (3.5)	92 (3.4)
Diabetes	845 (10.6)	176 (11.5)	150 (13.2)	298 (11.1)
Heart failure	72 (0.9)	23 (1.5)	25 (2.2)	38 (1.4)
Hypertension	1776 (22.3)	372 (24.3)	297 (26.2)	663 (24.7)
Ischemic heart disease^b^	361 (4.5)	81 (5.3)	58 (5.1)	143 (5.3)
Pneumonia	148 (1.9)	34 (2.2)	35 (3.1)	74 (2.8)
Any pulmonary condition other than ILD^b^	600 (7.5)	162 (10.6)	112 (9.9)	246 (9.2)
Scleroderma	15 (0.2)	3 (0.2)	7 (0.6)	2 (0.1)
Sjögren’s	121 (1.5)	32 (2.1)	19 (1.7)	51 (1.9)
SLE	122 (1.5)	26 (1.7)	37 (3.3)	52 (1.9)
Deyo-adapted CCI, mean (SD)^f^	1.5 (1.0)	1.6 (1.1)	1.6 (1.2)	1.6 (1.0)

*ILD* interstitial lung disease, *TNF* tumor necrosis factor, *SD* standard deviation, *RA* rheumatoid arthritis, *DMARD* disease-modifying anti-rheumatic drug, *NSAID* non-steroidal anti-inflammatory drug, *COPD* chronic pulmonary disease, *SLE* systemic lupus erythematosus, *CCI* Charlson comorbidity index

^a^ Demographics measured at study index

^b^ *P* < 0.05, tocilizumab versus all other cohorts combined

^c^ Using available data

^d^ In the 6 months prior to study index

^e^ Prednisone equivalents

^f^ In the 12 months prior to study index

There was considerable variability in prior exposure to RA medications across cohorts. Mean (SD) number of unique biologic agents received prior to index was significantly (*P* < 0.0001) higher among tocilizumab users (mean, 2.1; SD, 1.1) and lower among users of anti-TNF agents (mean, 1.4; SD, 0.7). Half of tocilizumab users had prior exposure to at least two biologics, and 25 % had exposure to at least three agents. In contrast, half of anti-TNF users had exposure to one prior biologic, and 25 % had exposure to at least two. Half (53.0 %) of patient episodes involved exposure to methotrexate in the 6 months prior to index, and 33 % were exposed to another non-biologic disease-modifying anti-rheumatic drug other than methotrexate; 42 % had recent exposure to prescription non-steroidal anti-inflammatory drugs. Recent exposure to glucocorticoids was highest among rituximab (81.1 %) and tocilizumab (80.4 %) episodes and lowest among anti-TNF agent (71.0 %) episodes (*P* < 0.0001). Tocilizumab and rituximab episodes not only were more likely to have had exposure to glucocorticoids but also were significantly (*P* < 0.0001) more likely to receive higher mean daily dosages (at least 7.5 mg/day) than were anti-TNF or abatacept episodes.

A little more than 8 % of the population had a history of some other form of pulmonary disease, primarily asthma (4.0 %), COPD (3.0 %), or pneumonia (2.2 %). Tocilizumab, rituximab, and abatacept episodes were more likely to have one of these pulmonary conditions (10.6 %, 9.9 %, and 9.2 % versus 7.5 %; *P* < 0.05) in the 12 months prior to index compared with anti-TNF treatments. Despite these differences, there was no statistically significant difference in the Deyo-adapted Charlson comorbidity index across cohorts. Baseline evidence of scleroderma, Sjögren’s, and systemic lupus erythematosus (SLE) occurred in 0.2 %, 1.7 %, and 1.8 % of episodes, respectively. There were no significant differences in the occurrence of these conditions across cohorts.

#### Patients with a baseline history of ILD

Table 2 depicts patient characteristics for patients with a history of ILD. One fifth of all patients with a history of ILD also had a history of oxygen use during baseline, reflecting in part ILD history but also other pulmonary disorders, which occurred in 38 % of episodes. Patients with a history of ILD and exposure to tocilizumab or rituximab were more likely to have a baseline history of pneumonia (25.4 % and 24.2 % versus 15.1 %) and COPD (22.0 % and 27.3 % versus 17.7 %) compared with patients exposed to anti-TNF therapy. Patients with a history of ILD and exposure to rituximab were more likely to have a recent (12 months prior to index) hospitalization for ILD, pneumonia, asthma, or COPD.

**Table 2:** Baseline demographics and clinical characteristics: patients with ILD history using the most sensitive ILD definition

	New anti-TNF Treatment episodes	New tocilizumab Treatment episodes	New rituximab Treatment episodes	New abatacept Treatment episodes
	N = 232	N = 59	N = 99	N = 109
Female, n (%)^a^	172 (74.1)	47 (79.7)	75 (75.8)	82 (75.2)
Age, mean (SD)^a^	60.4 (11.9)	59.4 (12.3)	62.1 (11.0)	61.2 (10.3)
Geographic region, n (%)^a^				
Northeast	36 (15.5)	9 (15.3)	13 (13.1)	15 (13.8)
North Central	73 (31.5)	16 (27.1)	42 (42.4)	35 (32.1)
South	78 (33.6)	26 (44.1)	24 (24.2)	43 (39.4)
West	43 (18.5)	8 (13.6)	20 (20.2)	15 (13.8)
Rural indicator, n (%)^a^	32 (13.8)	6 (10.2)	18 (18.2)	20 (18.3)
Medicare primary payer, n (%)^a^	78 (33.6)	17 (28.8)	43 (43.4)	35 (32.1)
Follow-up person years per patient, mean (SD)	0.7 (0.5)	0.6 (0.5)	0.7 (0.5)	0.7 (0.6)
No of prior biologics, mean (SD)^b,c^	1.6 (0.9)	2.3 (1.2)	1.7 (0.9)	1.6 (0.9)
Prior exposure to ≥2 biologics	45 (19.4)	30 (50.8)	27 (27.3)	10 (9.2)
Prior exposure to ≥3 biologics	12 (5.2)	6 (10.2)	2 (2.0	0 (0.0)
RA medication history, n (%)^d^				
Any anti-TNFα agent^b^	135 (58.2)	21 (35.6)	42 (42.4)	69 (63.3)
Methotrexate	73 (31.5)	25 (42.4)	39 (39.4)	44 (40.4)
All other DMARDs	108 (46.6)	21 (35.6)	52 (52.5)	54 (49.5)
Prescription NSAIDs	98 (42.2)	22 (37.3)	31 (31.3)	37 (33.9)
Glucocorticoid daily dosage, mean (SD)^e^				
None^b^	40 (17.2)	7 (11.9)	9 (9.1)	21 (19.3)
<7.5 mg/day	124 (53.4)	28 (47.5)	44 (44.4)	57 (52.3)
≥7.5 mg/day	68 (29.3)	24 (40.7)	46 (46.5)	31 (28.4)
Comorbid condition, n (%)^f^				
Anemia^b^	4 (1.7)	3 (5.1)	3 (3.0)	3 (2.8)
Asthma	23 (9.9)	5 (8.5)	12 (12.1)	5 (4.6)
Cerebrovascular disease	7 (3.0)	2 (3.4)	4 (4.0)	4 (3.7)
COPD^b^	41 (17.7)	13 (22.0)	27 (27.3)	18 (16.5)
Diabetes	41 (17.7)	16 (27.1)	20 (20.2)	25 (22.9)
Heart failure	12 (5.2)	5 (8.5)	8 (8.1)	12 (11.0)
Hypertension	72 (31.0)	20 (33.9)	34 (34.3)	36 (33.0)
Ischemic heart disease^b^	23 (9.9)	2 (3.4)	10 (10.1)	21 (19.3)
Pneumonia	35 (15.1)	15 (25.4)	24 (24.2)	17 (15.6)
Any pulmonary condition other than ILD^b^	81 (34.9)	26 (44.1)	50 (50.5)	32 (29.4)
Scleroderma	3 (1.3)	0 (0.0)	4 (4.0)	6 (5.5)
Sjögren’s	5 (2.2)	2 (3.4)	4 (4.0)	1 (0.9)
SLE	4 (1.7)	2 (3.4)	3 (3.0)	3 (2.8)
Any oxygen use, n (%)^f^	39 (16.8)	11 (18.6)	26 (26.3)	18 (16.5)
Hospitalization, n (%)^g^				
ILD	18 (7.8)	9 (15.3)	21 (21.2)	13 (11.9)
Pneumonia	24 (10.3)	8 (13.6)	18 (18.2)	12 (11.0)
Asthma	5 (2.2)	1 (1.7)	5 (5.1)	1 (0.9)
COPD	20 (8.6)	1 (1.7)	14 (14.1)	7 (6.4)
Deyo-adapted CCI, mean (SD)^f^	2.2 (1.4)	2.1 (1.0)	2.4 (1.4)	2.4 (1.6)

*ILD* interstitial lung disease, *TNF* tumor necrosis factor, *SD* standard deviation, *RA* rheumatoid arthritis, *DMARD* disease-modifying anti-rheumatic drug, *NSAID* non-steroidal anti-inflammatory drug, *COPD* chronic pulmonary disease, *SLE* systemic lupus erythematosus, *CCI* Charlson comorbidity index

^a^ Demographics measured at study index

^b^ *P* < 0.05, tocilizumab versus all other cohorts combined

^c^ Using available data

^d^ In the 6 months prior to study index

^e^ Prednisone equivalents

^f^ In the 12 months prior to study index

^g^ Based on the presence of a diagnosis code for the condition in any position on the claim. Hospitalization could occur at any point during the 12 months prior to study index, before, during or after diagnosis with ILD

It should also be noted that compared with patients without an ILD history, patients with a history of ILD were significantly more likely to receive rituximab (19.8 % versus 8.5 %; *P* < 0.0001) and significantly less likely to receive anti-TNF therapy (46.5 % versus 59.8 %; *P* < 0.0001). Although the frequency of scleroderma, Sjögren’s, and SLE was higher than was observed in the population with ILD history, occurring in 2.6 %, 2.4 %, and 2.4 % of episodes, respectively, there were no significant differences across cohorts.

### ILD incidence

Table 3 presents ILD incidence event counts and rates (95 % CI) for both specific and sensitive definitions. Overall, ILD incidence rate ranged from 1.8 per 1000 PY using the most specific definition to 6.4 using the most sensitive definition. Regardless of the definition employed, the vast majority (75 %–83 %) of events had at least one diagnosis code for post-inflammatory pulmonary fibrosis (ICD-9 CM 515.xx). Unadjusted ILD incidence (specific definition) rates, by cohort, ranged from a low of zero in the etanercept and golimumab cohorts to a high of 4.7 in the rituximab cohort. When the most sensitive definition was used, unadjusted ILD incidence rates ranged from a low of 4.0 in the abatacept cohort to a high of 12.2 (95 % CI 5.6–23.2) in the infliximab cohort. There was no statistically significant difference in the rate of ILD incidence, unadjusted, across cohorts.

**Table 3:** ILD incidence rate per 1000 PY, unadjusted

Cohort	Specific definition	Sensitive definition
	Events (total PY)	Events (total PY)
	Rate (95 % CI)	Rate (95 % CI)
All eligible	16 (9107)	59 (9154)
	1.8 (1.0–2.9)	6.4 (4.9–8.3)
Anti-TNFα agents	9 (5473)	39 (5527)
	1.6 (0.8–3.1)	7.1 (5.0–9.6)
Etanercept	0 (1012)	6 (1015)
	0.0 (0.0–3.0)	5.9 (2.2-12.9)
Adalimumab	3 (1674)	12 (1692)
	1.8 (0.4-5.2)	7.1 (3.7–12.4)
Infliximab	3 (735)	9 (738)
	4.1 (0.8–12.0)	12.2 (5.6–23.2)
Certolizumab pegol	3 (948)	7 (962)
	3.2 (0.7–9.3)	7.3 (2.9–15.0)
Golimumab	0 (1104)	5 (1119)
	0.0 (0.0–2.7)	4.5 (1.5–10.4)
Tocilizumab	1 (1008)	5 (1030)
	1.0 (0.0-5.5)	4.9 (1.6–11.3)
Rituximab	4 (851)	8 (830)
	4.7 (1.3–12.1)	9.6 (4.2–19.0)
Abatacept	2 (1775)	7 (1767)
	1.1 (0.1–4.1)	4.0 (1.6–8.2)

*ILD* interstitial lung disease, *PY* person-year, *CI* confidence interval, *TNFα* tumor necrosis factor alpha

**Fig. 2:**
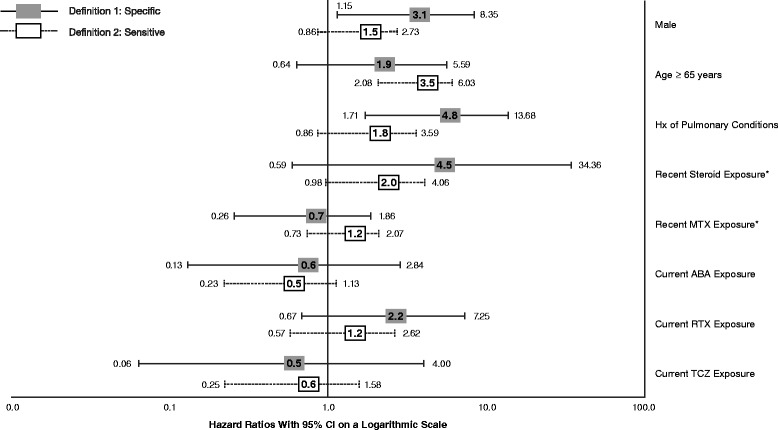
Relative hazard of ILD using the most specific and sensitive definitions. *ABA* abatacept, *Hx* history, *ILD* interstitial lung disease, *MTX* methotrexate, *RTX* rituximab, *TCZ* tocilizumab

Cox model results using both ILD definitions are presented in Fig. 2. When the most sensitive ILD definition was used, membership in the cohort of patients at least 65 years old was associated with increased risk of ILD (hazard ratio (HR) 3.54; 95 % CI 2.07–6.03; *P* < 0.0001), while recent exposure to glucocorticoids was associated with marginally increased risk (HR 1.99; 95 % CI 0.98–4.06; *P* = 0.0586). Cox model results using the most specific ILD definition suggested increased incidence associated with being male (HR 3.09; 95 % CI 1.14–8.35; *P* = 0.0258) and having a baseline history of other pulmonary conditions (HR 4.83; 95 % CI 1.71–13.68; *P* = 0.0030). No other variables were significant in this model. The relative hazard, compared with anti-TNF agents, associated with exposure to abatacept, rituximab, or tocilizumab was not significantly increased.

### ILD hospitalization

Table 4 presents ILD-related hospitalization event counts and rates (95 % CI) for both specific and sensitive definitions. ILD-related hospitalization rates ranged from 65.8 per 1000 PY to 127.7 using the specific and sensitive definitions, respectively. When either definition was used, the primary diagnosis for hospitalization was split evenly between ILD and pneumonia, with a single patient having a lung transplant. ILD-related hospitalization rates using the most specific definition ranged from a low of zero in each of the anti-TNF cohorts except etanercept to a high of 261.5 in the etanercept cohort. CIs using the specific definition are wide as would be expected, given the small number of events (n = 4). Unadjusted hospitalization rates using the most sensitive ILD definition ranged from a low of 55.6 in the tocilizumab cohort to a high of 262.5 in the infliximab cohort. There were no statistically significant differences in ILD-related hospitalization rates, unadjusted, across the study cohorts.

**Table 4:** ILD-related hospitalization rate per 1000 PY, unadjusted

Cohort	Specific definition	Sensitive definition
	Events (total PY)	Events (total PY)
	Rate (95 % CI)	Rate (95 % CI)
All eligible	4 (61)	42 (329)
	65.8 (17.9–68.4)	127.7 (92.0–172.6)
Anti-TNFα agents	1 (28)	17 (152)
	35.2 (0.9–195.9)	111.9 (65.2–179.1)
Etanercept	1	6 (33)
	261.5 (6.6–1456.7)	180.6 (66.3–393.1)
Adalimumab	0	3 (45)
	0.0 (0.0–253.9)	67.4 (13.9–197.0)
Infliximab	0	4 (15)
	0.0 (0.0–1445.4)	262.5 (71.5–672.2)
Certolizumab	0	2 (28)
	0.0 (0.0–761.4)	72.2 (8.7–260.7)
Golimumab	0	2 (31)
	0.0 (0.0–439.8)	63.9 (7.7–230.8)
Tocilizumab	1	2 (36)
	128.7 (3.3–717.1)	55.6 (6.7–200.7)
Rituximab	1 (13)	16 (68)
	78.8 (95 % CI 2.0–439.1)	234.2 (95 % CI 133.8–380.3)
Abatacept	1	7 (73)
	83.8 (95 % CI 2.1–467.0)	96.5 (95 % CI 38.8–198.7)

*ILD* interstitial lung disease, *PY* person-year, *CI* confidence interval, *TNFα* tumor necrosis factor alpha

**Fig. 3:**
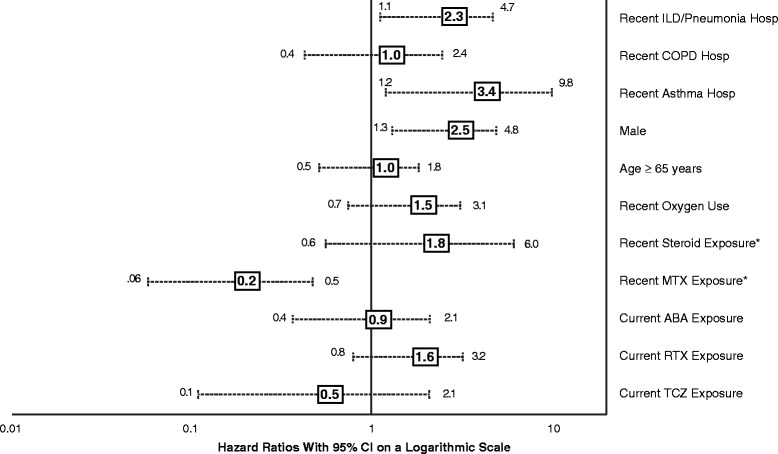
Relative hazard of ILD-related hospitalization using the most sensitive ILD definition. *ABA* abatacept, *COPD* chronic obstructive pulmonary disease, *hosp* hospitalization, *ILD* interstitial lung disease, *MTX* methotrexate, *RTX* rituximab, *TCZ* tocilizumab

Event counts were sufficient to support only a single Cox model assessing the relative hazard of an ILD-related hospitalization using the most sensitive definition. HRs and CIs are presented in Fig. 3. Recent exposure to methotrexate (HR 0.16; 95 % CI 0.06–0.46; *P* = 0.0007) was associated with a significant reduction in the risk of ILD-related hospitalizations, whereas being male (HR 2.47; 95 % CI 1.28–4.78; *P* = 0.0073) or having been hospitalized for asthma (HR 3.42; 95 % CI 1.19–9.82; *P* = 0.0224) or ILD/pneumonia (HR 2.28; 95 % CI 1.11–4.67; *P* = 0.0245) in the 12 months prior to index was significantly associated with an increased risk.

## Discussion

Few studies have examined the risk of incident ILD among patients exposed to anti-TNFs, abatacept, rituximab, and tocilizumab, and even fewer studies have evaluated the risk of hospitalization in patients with pre-existing ILD who are subsequently exposed to these agents. Our study evaluated both outcomes in a large cohort of RA patients exposed to biologic therapies and found no significant difference in the risk of ILD incidence between patients exposed to tocilizumab, rituximab, or abatacept compared with anti-TNFα therapies. In addition, no differences in the risk of ILD complications between patient cohorts exposed to each of the biologic agents were observed. Study findings confirmed that, in a real-world setting, there were significant baseline differences in patients exposed to alternate MOA as compared with patients exposed to anti-TNFs. Patients likely cycle between anti-TNFs before being given the therapeutic option of tocilizumab, rituximab, or abatacept. As a result, patients exposed to alternate MOA may have greater disease severity and duration. Physician perceptions may also play a role in channeling patients to and away from these newer therapies. Using data derived from administrative claims may not fully adjust for these differences, and any bias resulting from incomplete adjustment for confounding will bias the results in favor of anti-TNF therapies.

The proportion of patients with newly diagnosed ILD in our study ranged from 0.1 % to 0.4 %, depending on the ILD definition employed. Similarly, ILD incidence rates per 1000 PY ranged from 1.8 to 6.4. The frequency of ILD complications or exacerbation in patients with pre-existing ILD occurred in 4.1 %–8.4 % of patients at rates between 65.8 and 127.7 per 1000 PY. The incidence rates observed in the present study are similar to those previously reported, despite a markedly shorter follow-up window. Suissa et al. reported an ILD incidence rate of 0.81 per 1000 PY in their 2006 study assessing the impact of leflunomide on ILD risk [22]. Herrinton et al., in their 2013 evaluation of methotrexate and anti-TNFα agents, reported an age/sex-standardized ILD incidence rate, per 1000 PY, of 2.1 (95 % CI 0.0–4.3), with incident ILD observed in 0.8 % of patients with RA treated with an anti-TNF agent and 0.7 % in the methotrexate step-up cohort [19]. Dixon et al. [14] observed incident ILD in 2.8 % of patients receiving anti-TNFα agents, whereas Koike et al. [23] and Takeuchi et al. [24] reported rates of 0.6 % and 0.5 %, respectively.

The present study found that older patients, men, and patients with a history of other pulmonary disorders or recent exposure to glucocorticoids were at increased risk for developing ILD. Exposure to T-cell, B-cell, and IL-6 inhibitors was not significantly associated with increased ILD risk compared with exposure to an anti-TNFα agent. These data are largely consistent with those of the existing literature. Herrinton et al., in a cohort of patients with autoimmune disease, including RA, found that the risk of ILD was not increased in patients with exposure to anti-TNF therapy versus exposure to non-biologic disease-modifying anti-rheumatic drugs. Wolfe et al. estimated ILD risk by using inpatient and death record data, concluding that the only current treatment associated with hospital-associated ILD was glucocorticoids, although past exposure to a number of biologic agents (e.g., infliximab and etanercept) was associated with increased risk of ILD hospitalization [25]. Bongartz et al., using data from medical records, matched a cohort of patients with RA to those without the disease and found that the risk of developing ILD was higher in RA patients who were older at the time of disease onset, in male patients, and in individuals with more severe RA. The authors did not include biologic exposure in the analysis, but multivariate results did find that a history of methotrexate or glucocorticoids was associated with increased risk [26]. Chen et al. used high-resolution CT and pulmonary function tests (PFTs) to identify asymptomatic, preclinical forms of RA-ILD that may represent precursors to more severe fibrotic lung disease and concluded that patients with RA-ILD were older and had longer disease duration, higher articular disease activity, and more significant PFT abnormalities [27]. Several other studies have also found increased ILD risk associated with older age, male gender, and high levels of disease activity [23, 28–31].

ILD period prevalence in the present study was 4.2 %, which is within the range of previously published estimates. In a recent review of RA-associated ILD, O’Dwyer et al. described the evolution of ILD prevalence estimates beginning with Stack’s 1965 study based on chest radiographs (5 %), Frank’s 1973 study based on diffusion capacity (41 %), and Suzuki’s 1994 autopsy-based study (33 %) and concluding with the Bongartz [27] study that estimated 10-, 20-, and 30-year cumulative ILD incidence based on clinical data, PFT results, radiological studies, and lung biopsies [26]. The ranges reported by Bongartz et al. (3.5 %–7.7 %) are similar to those of Turesson et al., who conducted a similar study and reported a 30-year cumulative incidence for pulmonary fibrosis of 6.8 % [26, 32]. In addition, Olson et al. reported the prevalence of clinically significant ILD, defined as the presence of ILD in RA-associated deaths, to be approximately 6.8 % in women and 9.8 % in men [31]. The lifetime risk of developing ILD in patients with RA is now thought to be approximately 10 % [26].

Complications or exacerbation of ILD can be difficult to determine in administrative data. The present study used hospitalization for ILD, pneumonia, or lung transplant as a proxy for ILD exacerbation, observing that these complications occurred infrequently (4.1 %–8.4 %). Among patients with existing ILD, being male and having had a recent hospitalization for asthma, ILD, or pneumonia in the 12 months prior to index were associated with increased risk of an ILD-related hospitalization, whereas exposure to methotrexate in the 6 months prior to index was associated with significant reduction in an ILD-related hospitalization. Although methotrexate may in fact have a protective effect with respect to ILD exacerbation, these results are perhaps more likely to reflect channeling of patients with aggressive or severe ILD away from methotrexate because these patients have less pulmonary reserve were they to develop methotrexate-associated pneumonitis.

Although several studies have examined mortality in RA patients with ILD [14, 31, 33], we were able to identify only one study that reported the overall incidence of ILD exacerbation and its risk factors. Hozumi et al., in a 2013 study in Japan, reported the overall 1-year incidence of acute exacerbation of ILD in patients with RA at 2.8 % [8]. The authors further concluded that older age at ILD diagnosis, the usual interstitial pneumonia pattern on high-resolution CT, and methotrexate usage were associated with the development of acute exacerbation.

In an interpretation of our findings, several factors should be considered. Although the present study was based on a comparatively large and diverse sample of patients with RA, it was not a random population sample. The MarketScan databases are composed primarily of employer-sponsored coverage for active employees, dependents, and retirees. The prevalence of ILD or its complications might differ among those covered by other payers or the uninsured. Moreover, limited clinical information is available in these databases other than the diagnostic information recorded to support the claims for reimbursement. Patients with ILD were identified according to ICD-9-CM codes and were not confirmed by review of lung biopsy or CT results. In addition, whereas considerable literature details differences in ILD definition and detection, few studies have attempted to validate an algorithm for robust detection of this disease [19].

Since patients likely cycle between anti-TNFs before being given the therapeutic option of an alternate MOA, patients exposed to tocilizumab, rituximab, or abatacept may have greater disease severity and duration. Because options for adjusting for RA disease severity are limited in administrative claims, we selected a population of patients who had previously discontinued a different biologic agent prior to initiating the agents evaluated in this study. The incidence of ILD in individual exposure cohorts may still be confounded if patients with higher levels of disease severity were channeled to specific therapies. For example, prior biologic exposure and corticosteroid use was highest in patients in the tocilizumab and rituximab cohorts. Hospitalizations for asthma, COPD, ILD, and pneumonia were highest among patients with existing ILD subsequently initiating rituximab. To the extent that this is indicative of treatment resistance or disease severity, ILD incidence and exacerbation in the tocilizumab and rituximab cohorts may be overstated. In addition, given reports of rapid progression and increased mortality amongst users of anti-TNFα agents with existing ILD, it is also possible that clinicians are channeling patients away from anti-TNF therapy and toward other non-TNF biologics [14]. If such channeling exists, we expect the risk estimates for the non-TNF biologics to be greater than those observed with anti-TNFα agents.

Conversely, the observed ILD incidence rate in our study may have been suppressed by the limited length of the study follow-up window, although it should be noted that Roubille and Haraoui, in their systemic review of the literature, noted that ILD occurs mostly within the first 20 weeks after treatment initiation [18]. Similarly, Hadjinicolaou et al. summarized case reports in a review of the clinical trial literature and reported ILD cases occurring after relatively short exposures [15]. Finally, the numbers of events in both primary and secondary outcomes were small and, as such, may have constrained the identification of statistical significance.

## Conclusions

Baseline differences exist between RA patients receiving anti-TNFα agents and agents with alternate MOAs. Among patients with a history of biologic use, there were no significant differences in the risk of ILD and its related complications between RA patients receiving anti-TNFα agents and RA patients receiving T-cell, B-cell, and IL-6 inhibitors. Further studies are needed that account for clinical and baseline differences in order to fully evaluate risk of ILD and its complications in RA populations.

## Appendix

**Table 5:** Interstitial lung disease definition

ILD definition	ICD-9-CM code	ICD-9-CM description
Codes in the standard, specific definition of ILD	515.xx	Post-inflammatory pulmonary fibrosis
	516.30	Idiopathic interstitial pneumonia not otherwise specified
	516.31	Idiopathic pulmonary fibrosis
	516.32	Idiopathic non-specific interstitial pneumonitis
	516.33	Acute interstitial pneumonitis
	516.34	Respiratory bronchiolitis interstitial lung disease
	516.35	Idiopathic lymphoid interstitial pneumonia
	516.36	Cryptogenic organizing pneumonia
	516.37	Desquamative interstitial pneumonia
Additional codes added in the sensitive definition	516.8x	Other specified alveolar and parietoalveolar pneumonopathies
	516.9x	Unspecified alveolar and parietoalveolar pneumonopathy
	714.81	Rheumatoid lung

## Abbreviations

CI: Confidence interval
COPD: Chronic obstructive pulmonary disease
CT: Computed tomography
HCPCS: Health-care common procedure coding system
HR: Hazard ratio
IL-6: Interleukin-6
ILD: Interstitial lung disease
MOA: Mechanism of action
PFT: Pulmonary function test
PY: Person-years
RA: Rheumatoid arthritis
SD: Standard deviation
SLE: Systemic lupus erythematosus
TNF: Tumor necrosis factor
